# Factors associated with hand hygiene compliance at a tertiary care teaching hospital in Argentina

**DOI:** 10.1186/2047-2994-4-S1-P143

**Published:** 2015-06-16

**Authors:** RE Quirós, A Novau, D Latugaye, G Desmery

**Affiliations:** 1 Prevention and Infection Control Department, Hospital Universitario Austral, Pilar, Argentina

## Introduction

Several interventions have been implemented by hospitals to improve hand hygiene compliance. In that sense, the WHO Multimodal Hand Hygiene Improvement Strategy based on administrative support, system change including availability of alcohol-based handrub, education, reminders in the workplace, monitoring, and performance feedback has been successfully implemented.

## Objectives

To identify factors associated with hand hygiene compliance during a multiyear period of intervention.

## Methods

An observational prospective study including nursing, physician, technical, and support staff was conducted at a 142-bed tertiary care teaching hospital to assess those factors associated with hand hygiene compliance. Students from Nursing School performed hand hygiene observations through 21 cross-sectional studies from October 2009 to October 2014. Department for Infection Prevention and Control implemented a hospital-wide hand hygiene initiatives based on WHO Multimodal Hand Hygiene Improvement Strategy.

## Results

There were 27,484 unique observations with an overall compliance of 72.7% (95%CI 72.2%–73.2%). Significant differences in compliance were observed between nursing staff (80.2%; 95%CI 79.4%–80.9%), physician staff (69.5%; 95%CI 68.6%–70.49%) and support staff (64.5%; 95%CI 63.3%–65.6%). Neonatal Intensive Care Units (87.3%; 95%CI 85.7%–73.2%) and Bone Marrow Transplantation (82.2%; 95%CI 80.3%–83.9%) had higher compliance than did Adult Intensive Care Units (75.5%; 95%CI 74.6%–76.3%) and General Wards (66.1%; 95%CI 65.2%–67.0%). These findings persisted in the controlled multivariate model for compliance. Additional factor found to be significant in the model included greater compliance when healthcare workers were leaving patient rooms. The overall rate of compliance increased from 46.3% (95%CI 40.9%–51.7%) in the first year of observation to a peak of 79.6% (95%CI 78.5%–80.6%) in the fifth year, and it decreased to 77.7% (95%CI 76.7%–78.7%) in the final year.

## Conclusion

A Multimodal Hand Hygiene Improvement Strategy was effective in increasing compliance rates among all categories of hospital workers. We identified a variety of factors associated with increased compliance. Additionally, we note the importance of continuous interventions in maintaining high compliance rates.

## Disclosure of interest

None declared.

